# Metagenomic analysis of microbial communities in the sediments of a semi-intensive penaeid shrimp culture system

**DOI:** 10.1186/s43141-021-00237-9

**Published:** 2021-09-09

**Authors:** M. S. Chithira, P. V. Aishwarya, Anjali S. Mohan, Swapna P. Antony

**Affiliations:** Department of Marine Biology, Microbiology and Biochemistry, School of Marine Sciences, Cochin University of Science and Technology, Fine Arts Avenue, Kochi-16, Kerala India

**Keywords:** Metagenomics, Next-generation sequencing, 16S rRNA, Shrimp culture system, Sediment

## Abstract

The present study reports metagenomic sequencing and microbial diversity analysis of the sediment samples of a semi-intensive penaeid shrimp culture system. 16S rRNA gene-based high-throughput sequencing revealed distinct and diverse microbial communities in the analyzed sample. Analysis of the results showed a high abundance of Proteobacteria followed by Verrucomicrobia, Bacteroidetes, Planctomycetes, Firmicutes, Cyanobacteria, and Actinobacteria in the metagenome retrieved from the sediment sample. Unclassified bacteria also contributed a significant portion of the metagenome. Two potential shrimp pathogens viz *Vibrio harveyi* and *Acinetobacter lwoffii* detected in the sediment sample show the risk associated with the pond. Microbes that play essential roles in nutrient cycling and mineralization of organic compounds such as Bacteroidetes, Planctomycetes, Gammaproteobacteria, Firmicutes, Cyanobacteria, and Actinobacteria could also be identified. The present study provides preliminary data with respect to the microbial community present in the sediments of a shrimp culture system and emphasizes the application of metagenomics in exploring the microbial diversity of aquaculture systems, which might help in the early detection of pathogens within the system and helps to develop pathogen control strategies in semi-intensive aquaculture systems.

## Introduction

Aquaculture has grown rapidly during the last few decades due to research and developmental activities aiming at various aspects of aquaculture [1–3]. The use of microorganisms in aquaculture as environmental biomarkers, bioremediators, probiotics, and as a direct food source for the cultured species has expanded further in the last few decades [4–6]. However, we are still unaware of the various microbial species thriving within the aquaculture systems and their specific roles. Evidence has revealed that the diversity of microorganisms in aquaculture systems is far from being elucidated.

Metagenomics is the study of genetic material recovered directly from the environmental sample. It is a culture-independent approach that provides an ample opportunity to discover the unexplored microbial community [7]. Metagenomics undoubtedly can provide additional information regarding the understanding of the microbial diversity that thrives within the aquaculture systems. The present study reports metagenomic sequencing and analysis of the sediment samples of a semi-intensive penaeid shrimp culture system to explore its microbial diversity. 16S rRNA gene-based high-throughput sequencing was employed to reveal distinct and diverse microbial communities present in the sample.

## Material and methods

### Sample collection and processing

The present study was carried out in a semi-intensive aquaculture system for *Penaeus monodon* production, located at Puthuvype Kochi, Kerala, India (Lat: 9° 98′ 63′′ and Long: 76° 23′ 001′′). The aquaculture system operates under semi-intensive management, receiving natural water from the Cochin estuary. Approximately, 1.5 kg of sediment sample was collected from the culture pond from a depth of 80 cm by using a sterile grab. The sediment was black clayey with a salinity of 16 ppt and a temperature of 30°C. The sediment sample was immediately delivered on ice to the laboratory for immediate processing. Sediment was washed with wash buffer containing NaCl and Tween 20, followed by low-speed centrifugation at 700 rpm for 5 min. The supernatant was collected, which was again centrifuged at a high speed of 10000 rpm for 20 min. After centrifugation, the supernatant was subjected to multi-step filtration through a series of filter membranes with varied pore sizes including 11 μm, 1.45 μm, 0.45 μm, and 0.22 μm respectively for the removal of large sediment particles and other planktonic communities. The filter paper was then washed thrice with extraction buffer (1 M Tris-HCl, 0.5 M EDTA, 1 M Na_2_HPO_4_, 5 M NaCl) and kept on the rocker overnight. The bacteria-containing buffer was transferred to fresh vials and stored at −20 °C until further processing.

### Next-generation sequencing and bacterial diversity analysis

Metagenomic nucleic acid extracted from the sediment sample was subjected to 16S rRNA gene-based high-throughput sequencing and analysis at Clevergene Biocorp Pvt. Ltd., Bangalore, India. Briefly, 25 ng of DNA was used to amplify 16S rRNA hypervariable region V3–V4. The reaction included KAPA HiFi HotStart Ready Mix and 100 nm final concentration of modified 341-F and 785-R primers (F-CCTACGGGNGGCWGCAG; R-GACTACHVGGGTATCTAATCC) [8]. The PCR amplicons were purified using Ampure beads to remove unused primers. Additional eight cycles of PCR were performed using Illumina barcoded adapters to prepare the sequencing libraries. The sequence data quality was checked using FastQC and MultiQC software. The data was checked for base call quality distribution, % bases above Q20, Q30, % GC, and sequencing adapter contamination. The sample that passed the QC threshold (Q20 > 95%) was subjected to further analysis. The reads were trimmed and processed to remove degenerate primers. Adapter sequences and low-quality bases were removed, and sequence alignment was performed. The filtered contigs were processed and classified into taxonomical outlines based on the Greengenes v.13.8-99 database. The contigs were then clustered into operational taxonomic units (OTUs), and OTU abundance was estimated.

## Results and discussion

The microbial diversity is assumed to be greater in aquaculture systems due to the presence of nitrogenous and phosphorous metabolites as well as organic matter. Most of the microbial species thriving within the aquaculture systems and their specific roles still remain enigmatic. In this regard, metagenomics can provide additional information regarding the understanding of the microbial diversity that thrives within the aquaculture systems. The present study is a preliminary attempt to explore the microbial diversity present in the sediment of an aquaculture pond employing metagenomics.

**Fig. 1:**
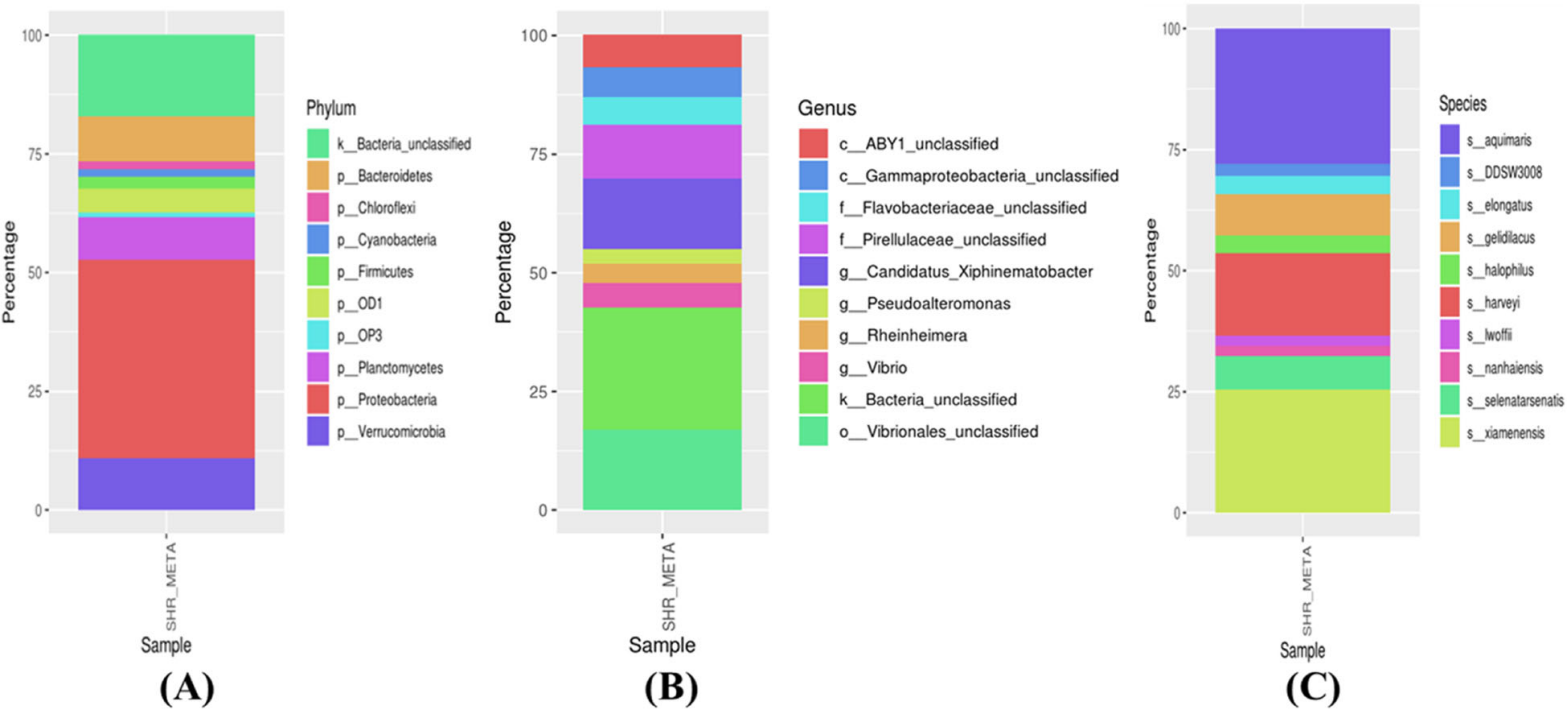
Diversity of microbial community identified using 16S rRNA gene-based high-throughput sequencing of the shrimp culture system. (**A**) Top 10 microbial community structure at the phylum level. (**B**) Top 10 microbial community structure at the genus level. (**C**) Top 10 microbial community structure at the species level

Next-generation sequencing of the sediment sample revealed distinct and diverse microbial communities present in the sample. Analysis of the results showed a high abundance of Proteobacteria in the metagenome retrieved from sediment sample followed by Verrucomicrobia, Bacteroidetes, Planctomycetes, Firmicutes, Cyanobacteria, and Actinobacteria. Figure 1 shows the relative abundance of the most dominant bacterial groups (ten most abundant phylum, ten most abundant genus, and ten most abundant species). Several unclassified bacteria could also be discovered from the sediment samples (Fig. 1). Alpha diversity metrics representing community richness indices (ACE and Chao1), and community diversity indices (Shannon, Simpson, InvSimpson, and Fisher) were also calculated.

Proteobacteria was found to be the most abundant phylum in the metagenome retrieved from the sediment sample. The present results agree with the reports of the metagenome analysis of a semi-intensive fish farm of European Sea bass [9], where also Proteobacteria was found to be the most abundant phylum. Proteobacteria play essential roles in nutrient cycling and mineralization of organic compounds and are found to be widely distributed in the marine environment [10, 11]. Previous studies have reported that proteobacteria dominate the gut microbiome of penaeid shrimps [10]. Proteobacteria have been reported to be more abundant in the shrimp intestines and are associated with slow growth performance and potential risk of disease. Some of the bacteria from this phylum are responsible for nitrogen fixation also. Most of the OTUs assigned to this phylum were assigned to be *Vibrio harveyi*, the potential shrimp pathogen that usually results in mass mortality. The high abundance of *V. harveyi* in the sediment shows the risk associated with the pond. *V. harveyi* is known to proliferate when there is excess organic matter in the sediment [12]. Another potential pathogen that could be detected in the sediment samples was *Acinetobacter lwoffii* which is also a proteobacterium.

The next abundant phyla of the sediment sample were Verrucomicrobia, capable of oxidizing a range of complex polymeric carbon compounds, enhancing the capacity of organic matter degradation in oxic sediments [13]. Bacteroidetes are a group of the intestinal microbiome that are beneficial to the host organism and abundant in the sediment samples. This phylum includes some of the most abundant groups in the marine systems after proteobacteria. Most of the OTUs assigned to this phylum were further classified as belonging to the class Flavobacteria and the order Flavobacteriales (Fig. 1). Flavobacteria are considered as potential bioremediators of the culture systems and play an important role in the degradation of organic matter [14, 15]. Species of the genus Bacteroidetes have been reported to show high antibiotic resistance capacity [16] and have been reported as a major vitamin B12 producer in the intestine of shrimps and finfishes [17]. Other abundant phyla identified in the sediment samples were beneficial bacteria belonging to Planctomycetes, Gammaproteobacteria, Firmicutes, Cyanobacteria, and Actinobacteria. These groups play a considerable role in the global carbon and nitrogen cycles and are involved in the bioremediation process. Many species of the phylum Planctomycetes are capable of anaerobic ammonium oxidation, also known as anammox. Among Planctomycetes, members of the family Pirullaceae, which are probiotic bacteria, were found to be dominating in the pond sediment.

Metagenomic analysis of the aquaculture systems will definitely pave way for elucidating the diversity of microorganisms present in the system and its potential role in the aquaculture system, including determination of metabolic processes performed by microbes; understanding the biogeochemical cycles of nutrients in the culture systems as well the development/outbreak of diseases.

In conclusion, taxonomic profiles of microbiotas in the sediment of shrimp farming environments were investigated in this study employing metagenomics. The present study provides preliminary data with respect to the microbial community present in the sediments of a semi-intensive shrimp culture system. Microbes are the most dominant group that harbors much in the sediments of shrimp ponds. The metagenomic analysis provides a better idea about the microbial communities present in an aquaculture system, especially the uncultivable ones. The present study emphasizes the application of metagenomics in exploring the microbial diversity of aquaculture systems, which might help detect pathogens within the system and helps to develop pathogen control strategies in the aquaculture systems.

## Data Availability

Not applicable.

## Abbreviations

DNA: Deoxyribonucleic acid
EDTA: Ethylenediamine tetraacetic acid
Na2HPO4: Sodium hydrogen phosphate
NaCl: Sodium chloride
OTUs: Operational taxonomic units
PCR: Polymerase chain reaction
rRNA: Ribosomal ribonucleic acid
Tris-HCl: Tris hydrochloride
